# Event and Comment

**Published:** 1921-12

**Authors:** 


### DENTAL REGISTER

THE MAGAZINE OF DENTISTRY

Vol. LXXV DECEMBER, 1921 No. 12

### EVENT AND COMMENT

**Technical Skill and its Limitations**

In every department of technical art and practice there is an increasing demand for all forms of technical skill; in fact it is greater than the profession can, under present conditions, take care of it properly.

We need only to mention the present demand for oral prophylaxis, pulp canal treatment, surgical extraction, and prosthodontia, to bring into review what is being attempted in these departments. We may mention a few of the other equally important, and even urgent subjects that in many cases are having only the most perfunctory considerations, or none at all, to remind us of the fact that we are getting behind with our work to such an extent that we are likely to lose out in trying to keep up with the pace which the general public expects us to maintain in the high standards which science and our art now demands of us.

The destruction of the natural teeth from dental caries and its affiliated diseases forces us to make new demands to eliminate all possible sources of infection from the oral system. This is now making a tremendous demand for all forms of artificial substitutes. This compels the prosthetists to renewed efforts to increase both the quantity and quality in this department. The better dentists have adopted a practice of doing every technical piece to as high a grade as science and skill can produce. While this is to be commended as a principle we find it difficult for the general practitioner to keep up a standard which is so exacting. The reason why equal standards are not practicable for all dentists and patients is obviously due to differences in skill and the ability of all patients to command the higher standards of skill because of increased costs. We find that prosthetic dentistry has not always appealed to the general practitioner. Traditionally it was practiced as a mechanical art by most dentists. It is only recently that true prosthodontia has made an appeal to the scientist as well as to the artisan. And we find that today the best educated and skilled dentists do insist that technical dentistry of every kind shall be practiced with the best skill and art that science knows. Therefore we have a continued loss in the number of skillful dentists because every dentist now is compelled to give more pains and thought to every patient he serves. Owing to the fact fewer men are studying dentistry today and each dentist serves fewer patients and our population is growing more numerous it follows that costs are higher.

We are glad to believe that more and more capable and devoted practitioners are giving attention to the prosthetic art, as there is a growing need which should be met in the best possible scientific and artistic way. We are already getting better results from this study and effort to give us more effective restorations for the serious losses of crippled dentitions because of the work of the extractors.

What we observe in connection with the practice of prosthodontia may be said with almost similar force about other branches of practice. The entire field of dentistry is being studied as never before, with great care and scientific accuracy. The fundamental sciences are being carefully studied because we have found out that theories that have only a basis of tradition upon which to build have not shown how to save the teeth from disease and loss. We must prove out our theories by the most exact and tried experiences.

We sometimes wonder if the tremendous upsetting of our moral standards incident to the great war may not become a real blessing, by creating more honest thinking and living the standards which in the future of mechanics and the arts, that we formerly leaned upon so trivially. It certainly would be great if our ethics could be of real practical service rather than things to dream about.

To change our technical formulae may be a more difficult problem than seems practicable. We are learning new truths all the while in the realms of science, and why should we not make some real discoveries in our art rather than remain content with things as they were done by our honored preceptors! Doctor Miller gave his life practically to learn what caused teeth to become carious. He discovered this truth but *we* have so far not followed up his clue and learned how to save the teeth from disease and loss. We shall need to do more studying and learn how to prevent dental caries by some scheme of practicable prophylaxis, or artistic reclaimation procedure, before we have completed the cycle of health and hygienic economics.

Evolution, and not revolution, must go on, especially for us, as our art and practice must be made safe and better scientifically.

Many things we did in the past we have had to change and remodel times without number because we were not wise enough to do the right things first; because we have learned most things by experimental rather than by scientific methods. Many things we must still do imperfectly because we don't know the truth, and we shall need still to practice tentatively until we arrive at the basic truths. We must know better than we do now what we can do that is scientifically practicable.

Our profession is now ready to say that all incurably diseased teeth should be extracted, because medical science has proved to us that there is serious danger from systemic infection by avleolar focal infections. Neverthless by force of traditional practice, and the reluctancy of patients to loose their teeth, science will have to make its demand forceful indeed to change the present methods of practice. The thought that we ultimately are destined to become edentulous if science forces this issue in unmistakable terms, is serious. Rather than give up all we have learned of curative dental art, we will be forced to learn at all hazards some scientific way of preventing dental caries. It would not be pleasant to look forward to living most of ones life on premasticated food. We are hopeful that dental hygiene has something to promise the future; and that prophylaxis and all forms of technical art are yet to be exhausted before we succumb to the exodontist. We trust we may not become weary in our quest for the truth, and hope that we shall find it in the fullness of time. In the meantime we must keep our minds and hearts in accord with the advancement of real medical science and the artistic practice of our profession. The best we have known is our technical art; and we must still cling to it until science makes a more satisfactory revelation.

